# Recent Advances in the Inhibition of the IL-4 Cytokine Pathway for the Treatment of Allergen-Induced Asthma

**DOI:** 10.3390/ijms222413655

**Published:** 2021-12-20

**Authors:** Oliver Massey, Cenk Suphioglu

**Affiliations:** 1 NeuroAllergy Research Laboratory, School of Life and Environmental Sciences, Deakin University, Geelong, VIC 3216, Australia; omassey@deakin.edu.au; 2 Institute for Mental and Physical Health and Clinical Translation (IMPACT), Deakin University, Geelong, VIC 3216, Australia

**Keywords:** interleukin 4 (IL-4), interleukin 13 (IL-13), immunoglobulin E (IgE), STAT6, immunotherapy, peptide therapeutics

## Abstract

The IL-4 and IL-13 cytokine pathways play integral roles in stimulating IgE inflammation, with the IL-4 cytokine being a major cytokine in the etiology of thunderstorm asthma, atopic dermatitis, and allergic rhinitis. The increasing prevalence of thunderstorm asthma in the younger population and the lessening efficacy of corticosteroids and other anti-inflammatories has created a need for more effective pharmaceuticals. This review summarizes the IL-4 and IL-13 pathways while highlighting and discussing the current pathway inhibitors aimed at treating thunderstorm asthma and atopic dermatitis, as well as the potential efficacy of peptide therapeutics in this field.

## 1. Introduction

Allergies are a worldwide issue, with approximately 20% of the global population suffering from allergy-caused symptoms including rashes (atopic dermatitis), runny nose (allergic rhinitis), and life-threatening breathing problems (allergy-induced asthma) [1]. This creates a gargantuan burden on global healthcare systems and lowers the quality of life for patients [1]. The term “allergy” was first described by Clemens von Piquet in 1903, wherein he determined that symptoms of disease were not solely caused by external infection, but also by the body’s response to said infection [2]. Since its conception, the term “allergy” has grown to describe a wide field within clinical immunology with even wider symptoms. One of the more serious symptoms of allergies is allergic asthma. Asthma currently affects as many as 339 million people globally, and is ranked 16th among the leading causes of years lived with disability and 28th among the leading causes of burden of disease [3]. The prevalence of asthma and allergies has greatly increased in recent decades, which can be attributed to a variety of factors, such as rapid urbanization and industrialization causing the release of air pollutants, smoking, exposure to mold, diet, and obesity, as well as genetic susceptibilities [3,4]. Additionally, it is still unknown whether the COVID-19 pandemic has had an effect on the prevalence of asthma; however, current reports from the United States suggest an increased risk of asthma in young people diagnosed with COVID-19 [5,6,7].

Asthma is commonly defined as smooth muscle contractions and chronic inflammation of the trachea and other associated airways, and can be caused by a variety of pathways involving gene and environmental interactions [8]. Research has revealed that over 100 genes are involved with pathways leading to asthma, but no single gene has been identified as the sole culprit, making treating asthma at its cause an arduous process [9]. Due to this, treatments for asthma have been heavily focused on the relief of symptoms on a day-to-day schedule, rather than addressing the underlying causes. These treatments are typically inhaled corticosteroids for short-term relief, or longer-lasting therapeutics such as long-acting β2–adrenergic receptors, leukotriene receptor antagonists, long-acting muscarinic antagonists, and for the most severe of symptoms, IgE-specific monoclonal antibody immunotherapy, also called omalizumab [10]. These treatments are all directed towards the relief of symptoms, rather than addressing the underlying pathways dysregulated in asthma. Of the standard therapeutic treatments, some are more successful in certain individuals than others. Certain trials have concluded that for 20% of their patients, asthma remained uncontrolled when treated with traditional corticosteroids [11].

The interleukin 4 and 13 pathways play integral roles in both type 1 allergy hypersensitivity and type 2 immunity [12,13]. Moderated by T-helper-2 lymphocytes, the IL-4 pathway causes the release of interleukin-4 from the aforementioned lymphocytes, which affects nearly every cell, while creating a variety of effects [14]. The IL-4 receptor is expressed on nearly every cell; however, due to the unique STAT6 molecule, the cellular response can differ between cells. For epithelial cells within the airways, cytokines IL-4 and IL-13 can cause muscle contraction and chronic inflammation, leading to asthmatic symptoms. Due to the integral role that the IL-4 pathway plays in allergen-induced asthma, it is a prime candidate for inhibitory drugs to treat allergic asthma.

## 2. IL-4 and IL-13 Pathways

Interleukins 4 and 13 are key cytokines in allergic inflammation, and are secreted by numerous immune cells, most commonly T-helper-2 lymphocytes [15]. The release of these cytokines causes the production of immunoglobulin E [IgE] by B lymphocytes, as well as causing a range of effects in other cells. The introduction of IgE to mast cells causes an immediate allergic inflammation and response. IL-4 plays another role in asthma through the induction of mucin gene expression, causing the hypersecretion of mucus from goblet cells within the airways. The IL-4 cytokine induces all these processes and more when released, causing the symptoms observed within allergic asthmatics.

When T-lymphocytes release IL-4, all cells carrying the receptor respond. While nearly all cells carry this receptor, overall cell responses differ between cell types due to the unique STAT6 transcription factor involved with this signaling pathway, which activates different intracellular transcription pathways dependent on that cell type. This allows for IL-4 to elicit a range of responses from a wide variety of cell types, making it the ideal candidate for therapeutic intervention. IL-13 differs from IL-4, as it is an effector cytokine that regulates smooth muscle contraction and mucus production. Similar to the IL-4 signaling system, IL-13 works extracellularly through cell membrane receptors.

The IL-4 signaling pathway involves numerous transmembrane peptides and intracellular signaling molecules, which produce unique responses dependent on cell type. The transmembrane receptor for IL-4 is IL-4Rα. IL-4Rα is expressed in most cells, although in low numbers. The role of this transmembrane peptide chain is to form a complex with IL-4, at which point it will recruit a secondary receptor chain. There are two secondary receptor chains, IL-2Ryc and IL-13Ra1, which are expressed within different cell types. Nonhematopoietic cells express IL-13Ra1, whereas IL-2Ryc is expressed in low levels or absent. The opposite of this is true for lymphocytes, which express IL-2Ryc, but little to no IL-13Ra1. The receptor complex formed between IL-4Rα and IL-2yc is herein referred to as the type 1 IL-4 complex, whereas the complex formed by IL-4Rα and IL-13Rα1 is herein referred to as the type 22 IL-4 receptor complex. Once the three signaling molecules have been recruited, the receptor has formed a functional complex, which then undergoes a conformational change, allowing for the activation of intracellular signaling machinery [16,17].

Once the receptor complex is formed and has undergone the conformational change to be functional, associated intracellular signaling molecules will undergo auto- and cross-phosphorylation, resulting in their activation. The intracellular machinery activated is largely dependent on the third signaling chain recruited to the transmembrane complex, although they all belong to a signaling family called the Jak kinases. The activation of these Jak kinases causes the phosphorylation of other kinases and critical Y residues within the intracellular domains of IL-4Rα. Once phosphorylation has occurred, these Y residues function as “docking sites” for intracellular signaling molecules, most commonly STAT6. STAT6 is an intracellular signaling molecule that grants the IL-4 pathway the unique property of producing different cellular responses dependent on cell type. Upon contact with the activated IL-4 signaling complex, STAT6 molecules homodimerize and translocate to the nucleus, binding to specific DNA sequences to influence the cells’ overall expression. The IL-4 signaling pathway is not only involved with transcriptional change, but is also involved with the downregulation of elicited signals. Pathways that downregulate signaling pathways are increased by IL-4 activation, limiting the duration of the IL-4 and associated signals.

**Figure 1:**
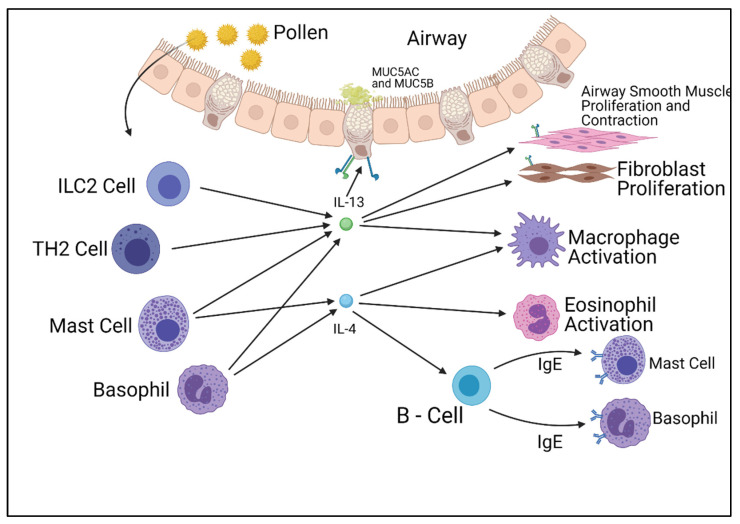
Summary of the cells responsible for the production of IL-4 and IL-13 and their effects on other cell types. Figure was created by the authors and reproduced with permission from BioRender.com (accessed on 19 November 2021).

The IL-13 pathway shares many functional similarities with the IL-4 pathway. IL-13 has two receptors similar to the IL-4 pathway, but differs by utilizing two separate binding chains. The type of receptor that is formed is dependent on which chain the IL-13 molecule binds to, unlike IL-4, which is determined after binding. The IL-13 pathway also affects the IL-4 pathway, as studies have displayed the complex formed by IL-13 and its type 1 alpha receptor (IL-13Rα1) lessen the intracellular effect of the type 2 IL-4 receptor complex, therefore reducing the cellular response. The IL-13 pathway concludes in the activation of STAT6 molecules and insulin receptor substrates (IRSs), which produce cellular responses at a transcription level. Unlike the IL-4 pathway, the IL-13 pathway is a poor activator for IRS molecules. Whilst STAT6 activates allergy symptoms, IRSs are involved with cellular proliferation. Both the IL-4 and IL-13 pathways play roles in the inflammation associated with allergies; however, they also play fundamental roles in cellular function [18,19,20] (Figure 1).

STAT6 is activated by both the IL-4 and IL-13 pathways, though this is largely dependent on the cell type, as are the effects of the STAT6 molecule. IL-4, however, can induce further intracellular signaling pathways, including Sos/Ras, PI3K/Akt, PKB/mTOR, and PKC. These alternative pathways, coupled with the transcriptional influence of STAT6, create variable cellular responses to the introduction of the IL-4 or IL-13 cytokines. While having effects on many different cell types, the main functions of the IL-4 and IL-13 pathways are the production of human immunoglobulin E and T-helper cell differentiation, which then brings on the clinical symptoms of atopic dermatitis, allergic rhinitis, and allergic asthma.

**Figure 2:**
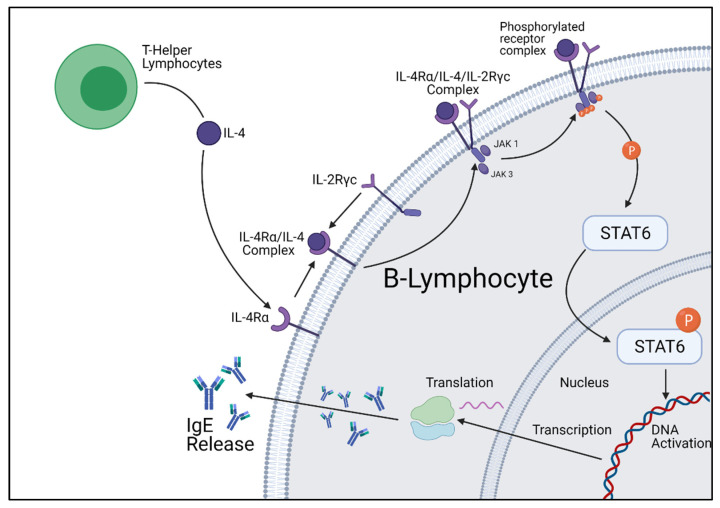
Cellular summary of B-lymphocyte IgE production initiated by IL-4 stimulation. Figure was created by the authors and reproduced with permission from BioRender.com (accessed on 19 November 2021).

STAT6 is a complex transcription factor responsible for exerting the effects of IL-4 and IL-13. Following phosphorylation by IL-4 and IL-13 receptor kinases, the phosphorylated STAT6 protein migrates to the nucleus to act as a transcription activator. It has been proven through mice knockout studies that STAT6 is required for allergy development; however, the genetic profile activated varies greatly between cell types. When STAT6 is activated within B-lymphocyte cells, STAT6 upregulates the Igε heavy chain and CD23 gene, which encodes for the IgE heavy chain and IgE receptors [21,22,23]. However, in Th2 cells, STAT6 upregulates gata3, another transcription factor responsible for many biological functions, and CRTH2, a gene involved in eosinophilic and allergic inflammation [24] (Figure 2).

## 3. Other Interleukin Pathways

IL-4 and IL-13 are not the sole interleukin cytokines responsible for airway inflammation. Whilst IL-4 and IL-13 cause the release of IgE and proinflammatory molecules from B-lymphocytes, proinflammatory molecules released from eosinophils play a pivotal role in causing airway inflammation and asthmatic symptoms [25]. Eosinophilic inflammation is a hallmark of all forms of asthma, and is a consequence of the uncontrolled production of IL-4, IL-13, and IL-5 [26]. IL-5 is the strongest activator of eosinophilic inflammation, capable of stimulating both eosinophil production and mast cell activation [27]. It is important to note that allergic reactions are a complex series of reactions involving intercellular communication. IL-4 and IL-13 are not the sole pathways responsible for the aforementioned allergy symptoms; however, their inhibitions have proven to be effective treatments.

## 4. IL-4 Pathway Inhibition

Due to their integral role in the production of IgE, the IL-4 and IL-13 cytokine pathways have been investigated as a target for potential therapeutic intervention. There exist a number of compounds that inhibit aspects of these cytokine pathways, although the only one that has been developed into a therapeutic that is approved for clinical use is dupilumab, a monoclonal antibody therapy that targets the IL-4 and IL-13 membrane receptors, specifically the alpha subunit of the IL-4Rα receptor. The IL-4Rα receptor is involved in both cytokine pathways, thereby making it the prime target for therapeutic inhibition. Dupilumab is currently approved for clinical use in the treatment of severe atopic dermatitis, though this treatment is invasive, requiring regular subdermal injections. There have been investigations into the use of dupilumab for the treatment of asthma, displaying promising results [28]. In one such study, conducted by Castro et al. in 2018, severe asthmatic patients were treated with either dupilumab or a placebo. Their results displayed that dupilumab had a significant positive effect in the treatment of asthma, reducing hospitalization visits and asthmatic episodes [29]. With no adverse side effects observed, dupilumab has a promising future, with ongoing studies evaluating and confirming its safety [30]. While only preliminary studies, these trials further solidify that IL-4- and IL-13-related inflammation are directly linked to asthma, and can be a target for therapeutic intervention [31].

Another drug that targets the IL-4 pathway is pascolizumab. This therapeutic is a humanized monoclonal antibody that targets the IL-4 cytokine. Initial animal studies were very promising, displaying almost complete T-cell function inhibition. Studies around this drug showed that pascolizumab had a high binding affinity to IL-4 with a slow dissociation rate [32]. This was coupled with low toxicity and few side effects, although these effects did become more exaggerated after chronic use, due to the drug’s long half-life. Previous animal trials have been conducted to evaluate short- and long-term treatment safety. The results from these trials were very promising, with the only complication being the development of an anti-idiotypic response resulting in the rapid clearance of pascolizumab [32]. In more recent years, a phase II clinical trial with pascolizumab was conducted with asthmatic patients. While no issues were raised with the safety of the treatment, the trials were discontinued due to the low efficacy [33].

Unlike the other therapeutics discussed, pitrakinra is a molecular therapeutic currently being investigated for IL-4 pathway inhibition. This peptide is a molecular inhibitor of the IL-4Rα surface receptor, competing for binding against both IL-4 and IL-13 [34]. Previous trials have shown pitrakinra having a low effect; however, as the trials progressed, pitrakinra began showing a greatly increased efficacy [35]. After further investigation, this variation was explained through genetic sequence variations between the patient groups, specifically a single nucleotide polymorphism within the IL-4Rα gene [36]. Results showed that patients carrying this SNP had a higher success rate than those without. Currently, pitrakinra is being further investigated for therapeutic effects within trials. Pitrakinra is a prime example of the role that genetics play in pharmaceuticals, displaying how a single nucleotide polymorphism can greatly affect the efficacy of a therapeutic. Further phenotypic investigations of the IL-4 and IL-13 pathways are required; this could allow for the subgrouping of patients by their genetic variations, opening the way to personalized therapeutic inhibitions.

## 5. IL-13 Inhibition

The IL-13 pathway, as discussed earlier, plays an integral role in type 1 inflammation, thereby making it a candidate for therapeutic inhibition. There are a number of therapeutic compounds, mostly monoclonal antibodies, that are being evaluated for potential clinical use in IL-13 inhibition (Table 1). Lebrikizumab is the first of these monoclonal antibodies that will be discussed in this review. This antibody inhibits the IL-13 pathway through binding to soluble IL-13, thereby preventing binding to the IL-13 cell surface receptors [37]. There have been a number of trials involving lebrikizumab that have shown positive effects in patients suffering from uncontrolled asthma [38]. While previous trials showed positive effects for asthmatic patients displaying type 1 biomarkers (high blood eosinophil count), a more recent phase II trial conducted in 2016 showed inconsistent reductions in asthma exacerbations, indicating further trials and studies are required to fully determine its efficacy in asthma treatment [39].

Anrukisumab is yet another monoclonal humanized anti-IL-13 antibody that inhibits the IL-13 pathway through binding to IL-13, preventing the formation of the receptor/ligand complex. Similarly to lebirkizumab, anrukisumab has been tested in clinical trials for the treatment of asthma, as well as for the treatment of ulcerative colitis [40,41]. This monoclonal antibody has, to date, only undergone phase I clinical trials aimed at toxicity; therefore, further studies are required to reach a better understanding of the underlying causes.

Tralokinumab is another humanized monoclonal antibody that also targets the interactions between IL-13 and its receptors. Unlike anrukisumab, tralokinumab has undergone phase II and III clinical trials aimed at finding a treatment for atopic dermatitis [42]. While aimed at atopic dermatitis, this is also relevant to allergen-induced asthma due to the integral role that IL-13 plays. Whilst having undergone a greater number of trials, tralokinumab showed similar flaws to other IL-13 inhibitors, such as inconsistent treatment across patient groups and low efficacy [43]. This demonstrated the intricate and numerous intertwining pathways that contribute towards allergic responses; while IL-4 and IL-13 pathways played integral roles, the pathways clearly varied among individuals. Very recently, phase III clinical trials have concluded for tralokinumab, and it has begun undergoing the review process for FDA approval, which was set to conclude in the second quarter of 2021. Similar to previous trials, the results of these later trials showed differing results between individuals, with some displaying a superior response to the treatment compared to others [44].

## 6. STAT6 Inhibition

Due to its integral role in both the Il-4 and Il-13 pathways, STAT6 has been previously investigated as a target for therapeutic inhibition. Experimental models have displayed that targeting the STAT6 transcription factor can inhibit airway inflammation, eosinophil infiltration, and fibrosis [45]. Due to this, a number of peptides have been identified that can bind to this transcription factor, thereby inhibiting its function, although these peptides have yet to be tested in vivo [46]. STAT6, while the prominent transcription factor for the production of IgE, has also been shown to play a number of roles throughout the body. For instance, it has been found that inhibition of STAT6 within gastric carcinoma cells reduced their protein expression, inhibiting their proliferation and migration [47]. Some studies have been performed examining the effect of various STAT6-inhibiting peptides, all with promising results showing decreased tumor growth and spread [48,49]; however, there are yet to be any in vivo preclinical or clinical trials, so the safety of these inhibitors is still relatively unknown.

## 7. Concluding Remarks

The overwhelming majority of inhibitory therapeutics currently being investigated for IL-4 pathway inhibition are immunotherapeutics that involve the use of large, monoclonal antibody molecules targeting either the IL-4 or IL-13 cytokines or their corresponding receptors. While targeting the same receptor or cytokine, these antibodies display different efficacy among patient groups, which can be attributed to small genetic variations, such as the 3′ genetic alteration discovered by the researchers studying lebrikizumab [39]. This emphasizes that more research is required into the minute variations between patient groups to determine the effect they have on treatment efficacy.

Despite targeting the same pathway, the aforementioned pharmaceutical trials displayed significantly different results for different patient groups. Further research into the small interpatient variations discussed previously could greatly increase the efficacy of these treatments while simultaneously opening the doors to personalized allergic treatments via cytokine pathway inhibition. Of the discussed inhibitory drugs, the majority are monoclonal antibody treatments, which carry their own sets of limitations. This type of immunotherapy has specific storage and clinical administration requirements, whilst also being expensive to manufacture [50]. They also require repeated administration when being used to treat autoimmune diseases, leading to high costs for patients and healthcare systems. As a proof of concept, these treatments have illustrated that the inhibition of the IL-4 and IL-13 pathways can effectively treat thunderstorm asthma, although costs and administration must improve before they can be considered as treatments.

Biologic treatment is a growing field offering highly specific and effective therapeutics. Whilst highly effective, biologics have a number of issues ranging from difficult production to invasive administration. Currently, there is one biologic treatment that targets the IL-4 pathway that has been approved, this being the aforementioned dupilumab. It is effective and can completely remove symptoms, although it is expensive and requires subepithelial administration through weekly injections [51]. High production costs and specialized invasive administration are the issues facing all biologic treatments [52,53]. Approaches have been made to deliver these therapeutics through alternative routes; however, there are many barriers. Biologics cannot be administered orally, as the digestive tract breaks down larger proteins and prevents their crossing of gastrointestinal epithelial membranes [54]. Similarly, most biologics are too large to cross the airway epithelium, thereby eliminating that route. Extensive research has been conducted to deliver biologics orally that involve altering and adding molecules to the surface of biologics, allowing them to retain their forms through the gastrointestinal tract and cross the epithelial membranes, although with such a wide variety of biologics, more research is required for better administration [55]. Smaller biological molecules are easier to administer, due to their increased stability and easier crossing of the epithelial membranes, which should be the target for future research.

This review has highlighted the interleukin 4 and 13 signaling pathways with current therapeutic inhibitors. Of the discussed inhibitors, the majority are immunotherapeutic, such as monoclonal antibodies that target either the cytokine or the receptor. These immunotherapies have their own limitations, as well as varying efficacy among patients, highlighting the need for further phenotyping of patient groups. Ideally, a therapeutic that targets the interleukin cytokine pathways should be easily transportable, noninvasive to administer, and show similar efficacy among patient groups. Due to the lack of success these immunotherapies have displayed against these criteria, perhaps research should focus on smaller, more stable molecules such as peptides or larger proteins, rather than complex immunological therapies.

## Figures and Tables

**Table 1:** Summary of IL-4 and IL-13 inhibitory drugs currently undergoing or that have undergone clinical trials.

Name	Inhibitory Target	Description	Reference
Dupilumab	IL-4Rα	Monoclonal antibody targeting the IL-14Rα receptor, thereby inhibiting both the IL-4 and IL-13 pathways. Currently approved in the US for treatment of atopic dermatitis. Under investigation for asthma treatment.	[29,30,31]
Pascolizumab	IL-4 cytokine	Humanized monoclonal antibody targeting the IL-4 cytokine. Binding to the cytokine inhibits prevents receptor binding, preventing the downstream effects of the IL-4 pathway.	[32,33]
Pitrakinra	IL-4α	Synthetic protein targeting the IL-4Rα receptor. Like dupilumab, pitrakinra inhibits both the IL-4 and IL-13 pathways, though clinical trials have shown little efficacy, leading to an investigation into the IL-4Rα genes.	[34,35,36]
Lebrikizumab	IL-13 cytokine	Monoclonal antibody that targets IL-13 cytokines, thereby blocking the downstream pathway. Trials are still being conducted, with inconsistent results reported.	[37,38,39]
Anrukisumab	IL-13 cytokine	Monoclonal antibody that also targets the IL-13 cytokine, like lebrikizumab, although it is aimed at the treatment of ulcerative colitis. Having only undergone phase I trials, more study is required.	[40,41]
Tralokinumab	IL-13	Monoclonal antibody that targets the IL-13 cytokine. Having recently undergone phase III trials, tralokinumab has shown promising results in the treatment of atopic dermatitis.	[42,43]
